# Antimicrobial activity of ceftazidime-avibactam and comparators against *Pseudomonas aeruginosa* and *Enterobacterales* collected in Croatia, Czech Republic, Hungary, Poland, Latvia and Lithuania: ATLAS Surveillance Program, 2019

**DOI:** 10.1007/s10096-022-04452-1

**Published:** 2022-05-20

**Authors:** V Adámková, I Mareković, J Szabó, L Pojnar, S Billová, S Horvat Herceg, A Kuraieva, B Możejko-Pastewka

**Affiliations:** 1 Institute of Medical Biochemistry and Laboratory Diagnostics, General University Hospital, Prague, Czech Republic; 2 Department of Clinical and Molecular Microbiology, University Hospital Centre Zagreb, Zagreb, Croatia; 3 Department of Medical Microbiology, Faculty of Medicine, University of Debrecen, Debrecen, Hungary; 4 Department of Microbiology, University Hospital, Cracow, Poland; 5 Pfizer, spol. s r.o., Prague, Czech Republic; 6 Pfizer Croatia, Zagreb, Croatia; 7 Pfizer Export B.V. RO, Kyiv, Ukraine; 8 Pfizer Polska Sp. z o.o, Warsaw, Poland

**Keywords:** Ceftazidime-avibactam, *Pseudomonas aeruginosa*, *Enterobacterales*, Antimicrobial surveillance, ATLAS, Difficult-to-treat

## Abstract

Antimicrobial susceptibility of clinical isolates collected from sites in central Europe in 2019 was tested by CLSI broth microdilution method and EUCAST breakpoints. Most active were amikacin, ceftazidime-avibactam and colistin; respectively, susceptibility rates among *P. aeruginosa* (n = 701) were 89.2%, 92.2% and 99.9%; difficult-to-treat (DTR) isolates, 62.5%, 37.5% and 100%; multidrug-resistant (MDR) isolates, 68.3%, 72.9% and 99.5%; meropenem-resistant (MEM-R), metallo-β-lactamase-negative (MBL-negative) isolates, 72.8%, 78.6% and 100%. Among *Enterobacterales* (*n* = 1639), susceptibility to ceftazidime-avibactam, colistin and tigecycline was ≥ 97.9%; MDR *Enterobacterales*, 96.8%, 94.4% and 100%, respectively; DTR isolates, ≥ 76.2% to ceftazidime-avibactam and colistin; MEM-R, MBL-negative isolates, ≥ 90.0% to ceftazidime-avibactam and colistin.

## Introduction

*Pseudomonas aeruginosa* and members of the *Enterobacterales* are important pathogens that cause a range of infections. Their treatment can be problematic due to acquired and/or intrinsic antimicrobial resistance [1, 2]. Ceftazidime (a third-generation cephalosporin) in combination with avibactam (a diazabicyclooctane, non-β-lactam, β-lactamase inhibitor) has activity against Gram-negative organisms with Ambler class A, class C and some class D (e.g. OXA-48 type) β-lactamases, although the combination is not active against class B metallo-β-lactamases (MBLs) [3–5].

ATLAS (Antimicrobial Testing Leadership And Surveillance) is a freely accessible antimicrobial surveillance program with a searchable online database (www.atlas-surveillance.com) designed to chart the in vitro activity of antimicrobial agents against Gram-positive and Gram-negative organisms collected globally. In this analysis, we evaluate the in vitro activity of ceftazidime-avibactam and comparator agents against *Pseudomonas aeruginosa* and *Enterobacterales* isolates collected in 2019 from patients in Croatia, Czech Republic, Hungary, Poland, Latvia and Lithuania.

## Materials and methods

Isolates of *P. aeruginosa* and *Enterobacterales* (*N* = 2340) were submitted by study centres in Croatia (*n* = 4), Czech Republic (*n* = 4), Hungary (*n* = 3), Poland (*n* = 4), Latvia (*n* = 1) and Lithuania (*n* = 2) in 2019 from patients of all ages. Acceptable sources were intra-abdominal, urinary tract, skin and skin structure, lower respiratory tract and bloodstream; only non-duplicate isolates of causative pathogens were accepted. Demographic information (specimen source, patient age and sex, and type of hospital setting) was recorded for each isolate.

Bacterial identification was confirmed at the central laboratory, International Health Management Associates, Inc. (IHMA; Schaumburg, IL, USA), using matrix-assisted laser desorption ionization-time of flight spectrometry (MALDI-TOF; Bruker Daltonics, Billerica, MA, USA). Susceptibility testing was according to the Clinical Laboratory Standards Institute (CLSI) broth microdilution methodology [6]. Ceftazidime-avibactam was tested with fixed concentration of avibactam at 4 mg/L. All minimum inhibitory concentration (MIC) values were interpreted using EUCAST breakpoints [7].

Difficult-to-treat (DTR) isolates were resistant to aztreonam, cefepime, ceftazidime, imipenem, meropenem, ciprofloxacin, levofloxacin and piperacillin-tazobactam. Multidrug-resistant (MDR) isolates were resistant to ≥ 1 agent from ≥ 3 classes: cephalosporins (ceftazidime, cefepime), monobactams (aztreonam), β-lactam/β-lactamase-inhibitor combinations (piperacillin-tazobactam), carbapenems (meropenem, imipenem), fluoroquinolones (levofloxacin, ciprofloxacin), aminoglycosides (amikacin) and polymyxins (colistin). Meropenem-resistant (MEM-R) isolates were isolates with an MIC to meropenem of ≥ 16 mg/L. Carbapenemase and metallo-β-lactamase (MBL) genes were determined using polymerase chain reaction (PCR) assays [8, 9]. Detected genes were amplified using flanking primers and sequenced, and sequences were compared against publicly available databases. Carbapenemase-positive isolates were identified as those with genes encoding a KPC, OXA-48-like, IMP, VIM, NDM, GES, GIM and/or SPM enzyme, and MBL-positive isolates were identified as those with genes encoding an NDM, IMP, VIM, GIM and/or SPM enzyme. MBL-negative isolates were defined as those that underwent testing but did not possess NDM, IMP, VIM, GIM and SPM genes.

## Results

The majority of *P. aeruginosa* (*n* = 701) and *Enterobacterales* isolates (*n* = 1639) were collected from male patients, patients ≥ 18 years of age and non-ICU wards (Table 1). The highest proportion of *P. aeruginosa* isolates were from respiratory sources. Similar percentages of *Enterobacterales* isolates were from blood, respiratory or skin/musculoskeletal sources (Table 1).

**Table 1:** Demographic data for *Pseudomonas aeruginosa* and *Enterobacterales* isolates, collected from Croatia, Czech Republic, Hungary, Poland, Latvia and Lithuania, 2019

	*Pseudomonas aeruginosa*		*Enterobacterales*	
	*N* = 701		*N* = 1639	
	*n*	%	*n*	%
Age groups (years)				
0 to 17	85	12.1	151	9.2
18 to 64	261	37.2	525	32.0
≥ 65	353	50.4	958	58.5
Unknown	2	0.3	5	0.3
Sex				
Female	246	35.1	678	41.4
Male	453	64.6	955	58.3
Unknown	2	0.3	6	0.4
Patient location				
ICU	271	38.7	511	31.2
General wards, Emergency	395	56.3	1043	63.6
Unknown/Other	35	5.0	85	5.2
Isolates sources				
Circulatory (blood)	114	16.3	403	24.6
Genitourinary	82	11.7	253	15.4
Intestinal	34	4.9	213	13.0
Respiratory	296	42.2	415	25.3
Skin/musculoskeletal	174	24.8	355	21.7
Unknown	1	0.1	0	0.0

### *Pseudomonas aeruginosa*

The agents against which *P. aeruginosa* had the highest rates of susceptibility (using standard dosing susceptibility breakpoints) were amikacin (89.2%), ceftazidime-avibactam (92.2%) and colistin (99.9%) (Table 2). For ceftazidime alone, 74.3% of isolates were susceptible (increased exposure). A total of 5.7% (40/701) of isolates were classified as DTR and 28.4% (199/701) were MDR. Among these isolates, susceptibility to colistin was unchanged (100% and 99.5%, respectively) relative to the whole *P. aeruginosa* set. Susceptibility rates to amikacin and ceftazidime-avibactam were 62.5% and 37.5%, respectively, against DTR isolates and 68.3% and 72.9%, respectively, against MDR isolates (Table 2). Results against MEM-R isolates were similar to those seen against MDR isolates for the majority of agents (Table 2). Against all three resistant subsets, rates of susceptibility (increased exposure) to ceftazidime (DTR, 0.0%; MDR, 16.1%; MEM-R, 24.0%) were lower than susceptibility rates (standard dosing) reported for ceftazidime-avibactam.

**Table 2:** Antimicrobial activity of ceftazidime-avibactam and comparators against *Pseudomonas aeruginosa* isolates collected from Croatia, Czech Republic, Hungary, Poland, Latvia and Lithuania in 2019

Antimicrobial	MIC_90_ (mg/L)	Range (mg/L)	Susceptible, standard dosing		Susceptible, increased exposure		Resistant	
*P. aeruginosa* (*n* = 701)			*n*	%	*n*	%	*n*	%
Amikacin	32	0.5 – ≥ 128	625	89.2	–	–	76	10.8
Aztreonam	32	0.25 – ≥ 256	–	–	586	83.6	115	16.4
Cefepime	32	0.5 – ≥ 64	–	–	539	76.9	162	23.1
Ceftazidime	64	0.25 – ≥ 256	–	–	521	74.3	180	25.7
Ceftazidime-avibactam	8	0.12 – ≥ 256	646	92.2	–	–	55	7.8
Ciprofloxacin	≥ 8	≤ 0.12 – ≥ 8	–	–	477	68.0	224	32.0
Colistin	2	0.25 – ≥ 16	700	99.9	–	–	1	0.1
Gentamicin	≥ 32	≤ 0.12 – ≥ 32	–	–	–	–	–	–
Imipenem	≥ 16	≤ 0.06 – ≥ 16	–	–	473	67.5	228	32.5
Levofloxacin	≥ 16	≤ 0.25 – ≥ 16	–	–	437	62.3	264	37.7
Meropenem	16	≤ 0.06 – ≥ 32	471	67.2	105	15.0	125	17.8
Piperacillin-tazobactam	≥ 128	≤ 0.12 – ≥ 128	–	–	501	71.5	200	28.5
Tigecycline	≥ 16	1 – ≥ 16	–	–	–	–	–	–
DTR *P. aeruginosa* (*n* = 40)								
Amikacin	64	2 – ≥ 128	25	62.5	–	–	15	37.5
Aztreonam	64	32 – ≥ 256	–	–	0	0.0	40	100
Cefepime	≥ 64	16 – ≥ 64	–	–	0	0.0	40	100
Ceftazidime	≥ 256	16 – ≥ 256	–	–	0	0.0	40	100
Ceftazidime-avibactam	≥ 256	4 – ≥ 256	15	37.5	–	–	25	62.5
Ciprofloxacin	≥ 8	1 – ≥ 8	–	–	0	0.0	40	100
Colistin	2	0.5 – 2	40	100	–	–	0	0.0
Gentamicin	≥ 32	0.25 – ≥ 32	–	–	–	–	–	–
Imipenem	≥ 16	8 – ≥ 16	–	–	0	0.0	40	100
Levofloxacin	≥ 16	4 – ≥ 16	–	–	0	0.0	40	100
Meropenem	≥ 32	16 – ≥ 32	0	0.0	0	0.0	40	100
Piperacillin-tazobactam	≥ 128	32 – ≥ 128	–	–	0	0.0	40	100
Tigecycline	≥ 16	1 – ≥ 16	–	–	–	–	–	–
MDR *P. aeruginosa* (*n* = 199)								
Amikacin	≥ 128	0.5 – ≥ 128	136	68.3	–	–	63	31.7
Aztreonam	64	4 – ≥ 256	–	–	88	44.2	111	55.8
Cefepime	≥ 64	2 – ≥ 64	–	–	45	22.6	154	77.4
Ceftazidime	≥ 256	2 – ≥ 256	–	–	32	16.1	167	83.9
Ceftazidime-avibactam	64	1 – ≥ 256	145	72.9	–	–	54	27.1
Ciprofloxacin	≥ 8	≤ 0.12 – ≥ 8	–	–	51	25.6	148	74.4
Colistin	2	0.25 – ≥ 16	198	99.5	–	–	1	0.5
Gentamicin	≥ 32	≤ 0.12 – ≥ 32	–	–	–	–	–	–
Imipenem	≥ 16	0.5 – ≥ 16	–	–	54	27.1	145	72.9
Levofloxacin	≥ 16	≤ 0.25 – ≥ 16	–	–	38	19.1	161	80.9
Meropenem	≥ 32	≤ 0.06 – ≥ 32	40	20.1	46	23.1	113	56.8
Piperacillin-tazobactam	≥ 128	8 – ≥ 128	–	–	20	10.1	179	89.9
Tigecycline	≥ 16	1 – ≥ 16	–	–	–	–	–	–
MEM-R *P. aeruginosa* (*n* = 125)								
Amikacin	≥ 128	1 – ≥ 128	82	65.6	–	–	43	34.4
Aztreonam	64	4 – ≥ 256	–	–	68	54.4	57	45.6
Cefepime	≥ 64	2 – ≥ 64	–	–	39	31.2	86	68.8
Ceftazidime	≥ 256	2 – ≥ 256	–	–	30	24.0	95.0	76.0
Ceftazidime-avibactam	64	2 – ≥ 256	82	65.6	–	–	43	34.4
Ciprofloxacin	≥ 8	≤ 0.12 – ≥ 8	–	–	23	18.4	102	81.6
Colistin	2	0.25 – 2	125	100	–	–	0	0.0
Gentamicin	≥ 32	0.25 – ≥ 32	–	–	–	–	–	–
Imipenem	≥ 16	1 – ≥ 16	–	–	2	1.6	123	98.4
Levofloxacin	≥ 16	0.5 – ≥ 16	–	–	11	8.8	114	91.2
Piperacillin-tazobactam	≥ 128	4 – ≥ 128	–	–	23	18.4	102	81.6
Tigecycline	≥ 16	1 – ≥ 16	–	–	–	–	–	–
MEM-R, MBL-negative *P. aeruginosa* (*n* = 103)								
Amikacin	64	1 – ≥ 128	75	72.8	–	–	28	27.2
Aztreonam	64	4 – ≥ 256	–	–	55	53.4	48	46.6
Cefepime	32	2 – ≥ 64	–	–	37	35.9	66	64.1
Ceftazidime	128	2 – ≥ 256	–	–	30	29.1	73	70.9
Ceftazidime-avibactam	16	2 – ≥ 256	81	78.6	–	–	22	21.4
Ciprofloxacin	≥ 8	≤ 0.12 – ≥ 8	–	–	18	17.5	85	82.5
Colistin	2	0.25 – 2	103	100	–	–	0	0.0
Gentamicin	≥ 32	0.25 – ≥ 32	–	–	–	–	–	–
Imipenem	≥ 16	1 – ≥ 16	–	–	2	1.9	101	98.1
Levofloxacin	≥ 16	0.5 – ≥ 16	–	–	8	7.8	95	92.2
Piperacillin-tazobactam	≥ 128	4 – ≥ 128	–	–	22	21.4	81	78.6
Tigecycline	≥ 16	1 – ≥ 16	–	–	–	–	–	–

MIC, minimum inhibitory concentration; DTR, difficult to treat; MDR, multidrug resistant; MEM-R, meropenem resistant; MBL, metallo-β-lactamase

Among the MEM-R *P. aeruginosa*, 82.4% (103/125) were identified as MBL-negative. All MBL-negative isolates were susceptible to colistin (Table 2), 78.6% to ceftazidime-avibactam and 72.8% to amikacin. A total of 29.1% of MBL-negative isolates were susceptible (increased exposure) to ceftazidime alone.

Among the 125 MEM-R isolates, 22 (17.6%) were MBL-positive and 29 (23.2%) were carbapenemase-positive. Colistin was the only agent active against the MBL-positive isolates (100% susceptible, data not shown).

### *Enterobacterales*

Susceptibility to amikacin, ceftazidime-avibactam, colistin and meropenem against *Enterobacterales* was ≥ 96.1%, and to ceftazidime alone, 69.5% (Table 3). Susceptibility to tigecycline was 99.8% (*E. coli* and *C. koseri*, only).

**Table 3:** Antimicrobial activity of ceftazidime-avibactam and comparators against *Enterobacterales* isolates collected from Croatia, Czech Republic, Hungary, Poland, Latvia and Lithuania in 2019

Antimicrobial	MIC_90_ (mg/L)	MIC range (mg/L)	Susceptible, standard dosing		Susceptible, increased exposure		Resistant	
*Enterobacterales* (*n* = 1639)			*n*	%	*n*	%	*n*	%
Amikacin	8	≤ 0.25 – ≥ 128	1575	96.1	–	–	64	3.9
Amoxicillin-clavulanate	≥ 32	≤ 0.12 – ≥ 32	923	56.3	–	–	716	43.7
Aztreonam	64	≤ 0.015 – ≥ 256	1157	70.6	39	2.4	443	27.0
Cefepime	≥ 64	≤ 0.12 – ≥ 64	1198	73.1	70	4.3	371	22.6
Ceftazidime	64	≤ 0.015 – ≥ 256	1139	69.5	52	3.2	448	27.3
Ceftazidime-avibactam	0.5	≤ 0.015 – ≥ 256	1626	99.2	–	–	13	0.8
Ciprofloxacin	≥ 8	≤ 0.12 – ≥ 8	1092	66.6	45	2.7	502	30.6
Colistin^a^	1	≤ 0.06 – ≥ 16	1304	97.9	–	–	28	2.1
Gentamicin	≥ 32	≤ 0.12 – ≥ 32	1314	80.2	–	–	325	19.8
Imipenem	2	≤ 0.06 – ≥ 16	1362	83.1	231	14.1	46	2.8
Levofloxacin	≥ 16	≤ 0.25 – ≥ 16	1170	71.4	87	5.3	382	23.3
Meropenem	0.12	≤ 0.06 – ≥ 32	1587	96.8	22	1.3	30	1.8
Piperacillin-tazobactam	≥ 128	≤ 0.12 – ≥ 128	1240	75.7	–	–	399	24.3
Tigecycline^b^	2	0.06 – 8	472	99.8	0	0.0	1	0.2
DTR *Enterobacterales* (*n* = 21)								
Amikacin	≥ 128	2 – ≥ 128	12	57.1	–	–	9	42.9
Amoxicillin-clavulanate	≥ 32	≥ 32	0	0.0	–	–	21	100
Aztreonam	≥ 256	16 – ≥ 256	0	0.0	0	0.0	21	100
Cefepime	≥ 64	32 – ≥ 64	0	0.0	0	0.0	21	100
Ceftazidime	≥ 256	32 – ≥ 256	0	0.0	0	0.0	21	100
Ceftazidime-avibactam	≥ 256	0.5 – ≥ 256	16	76.2	–	–	5	23.8
Ciprofloxacin	≥ 8	4 – ≥ 8	0	0.0	0	0.0	21	100
Colistin^a^	2	0.25 – ≥ 16	20	95.2	–	–	1	4.8
Gentamicin	≥ 32	0.5 – ≥ 32	8	38.1	–	–	13	61.9
Imipenem	≥ 16	≥ 16	0	0.0	0	0.0	21	100
Levofloxacin	≥ 16	2 – ≥ 16	0	0.0	0	0.0	21	100
Meropenem	≥ 32	≥ 32	0	0.0	0	0.0	21	100
Piperacillin-tazobactam	≥ 128	≥ 128	0	0.0	–	–	21	100
Tigecycline^b^	2	0.25 – 4	–	–	–	–	–	–
MDR *Enterobacterales* (*n* = 410)								
Amikacin	16	0.5 – ≥ 128	366	89.3	–	–	44	10.7
Amoxicillin-clavulanate	≥ 32	2 – ≥ 32	108	26.3	–	–	302	73.7
Aztreonam	≥ 256	0.03 – ≥ 256	5	1.2	6	1.5	399	97.3
Cefepime	≥ 64	≤ 0.12 – ≥ 64	35	8.5	39	9.5	336	82.0
Ceftazidime	≥ 256	0.25 – ≥ 256	5	1.2	12	2.9	393	95.9
Ceftazidime-avibactam	2	0.06 – ≥ 256	397	96.8	–	–	13	3.2
Ciprofloxacin	≥ 8	≤ 0.12 – ≥ 8	69	16.8	9	2.2	332	81.0
Colistin^a^	1	0.12 – ≥ 16	371	94.4	–	–	22	5.6
Gentamicin	≥ 32	≤ 0.12 – ≥ 32	180	43.9	–	–	230	56.1
Imipenem	4	0.12 – ≥ 16	347	84.6	26	6.3	37	9.0
Levofloxacin	≥ 16	≤ 0.25 – ≥ 16	120	29.3	45	11.0	245	59.8
Meropenem	4	≤ 0.06 – ≥ 32	358	87.3	22	5.4	30	7.3
Piperacillin-tazobactam	≥ 128	0.5 – ≥ 128	81	19.8	–	–	329	80.2
Tigecycline^b^	2	0.06 – 8	53	100	0	0.0	0	0.0
MEM-R *Enterobacterales* (*n* = 30)								
Amikacin	≥ 128	0.5 – ≥ 128	20	66.7	–	–	10	33.3
Amoxicillin-clavulanate	≥ 32	16 – ≥ 32	0	0.0	–	–	30	100
Aztreonam	≥ 256	0.25 – ≥ 256	2	6.7	0	0.0	28	93.3
Cefepime	≥ 64	2 – ≥ 64	0	0.0	1	3.3	29	96.7
Ceftazidime	≥ 256	0.5 – ≥ 256	1	3.3	0	0.0	29	96.7
Ceftazidime-avibactam	≥ 256	0.12 – ≥ 256	21	70.0	–	–	9	30.0
Ciprofloxacin	≥ 8	≤ 0.12 – ≥ 8	1	3.3	0	0.0	29	96.7
Colistin^a^	2	0.25 – ≥ 16	28	93.3	–	–	2	6.7
Gentamicin	≥ 32	0.25 – ≥ 32	13	43.3	–	–	17	56.7
Imipenem	≥ 16	2 – ≥ 16	2	6.7	2	6.7	26	86.7
Levofloxacin	≥ 16	0.5 – ≥ 16	1	3.3	3	10.0	26	86.7
Piperacillin-tazobactam	≥ 128	64 – ≥ 128	0	0.0	–	–	30	100
Tigecycline^b^	2	0.25 – 4	–	–	–	–	–	–
MEM-R, MBL-negative *Enterobacterales* (*n* = 20)								
Amikacin	≥ 128	0.5– ≥ 128	11	55.0	–	–	9	45.0
Amoxicillin-clavulanate	≥ 32	16– ≥ 32	0	0.0	–	–	20	100
Aztreonam	≥ 256	0.25– ≥ 256	1	5.0	0	0.0	19	95.0
Cefepime	≥ 64	2– ≥ 64	0	0.0	1	5.0	19	95.0
Ceftazidime	≥ 256	0.5– ≥ 256	1	5.0	0	0.0	19	95.0
Ceftazidime-avibactam	4	0.12– ≥ 256	19	95.0	–	–	1	5.0
Ciprofloxacin	≥ 8	4– ≥ 8	0	0.0	0	0.0	20	100
Colistin^a^	2	0.25– ≥ 16	18	90.0	–	–	2	10.0
Gentamicin	≥ 32	0.25– ≥ 32	7	35.0	–	–	13	65.0
Imipenem	≥ 16	2– ≥ 16	2	10.0	2	10.0	16	80.0
Levofloxacin	≥ 16	1– ≥ 16	0	0.0	1	5.0	19	95.0
Piperacillin-tazobactam	≥ 128	64– ≥ 128	0	0.0	–	–	20	100
Tigecycline^b^	2	0.25–4	–	–	–	–	–	–

MIC, minimum inhibitory concentration; ESBL, extended-spectrum β-lactamase; DTR, difficult to treat; MDR, multidrug resistant; MEM-R, meropenem resistant; MBL, metallo-β-lactamase

^a^For colistin, *Morganella morganii*, *Proteus* spp., *Providencia* spp. and *Serratia* spp. were excluded from analysis because of their intrinsic resistance; therefore, number of isolates tested against colistin: *Enterobacterales*, *n* = 1332; ESBL-positive *Enterobacterales*, *n* = 297; DTR *Enterobacterales*, *n* = 21; MDR *Enterobacterales*, *n* = 393; MEM-R *Enterobacterales*, *n* = 30; MBL-negative *Enterobacterales*, *n* = 20

^b^For tigecycline, susceptibility and resistance rates among the *Enterobacterales* were only calculated for *Escherichia coli* and *Citrobacter koseri* as EUCAST breakpoints are only approved against these species: *Enterobacterales*, *n* = 473; ESBL-positive *Enterobacterales*, *n* = 66; DTR *Enterobacterales*, *n* = 1; MDR *Enterobacterales*, *n* = 53; MEM-R *Enterobacterales*, *n* = 1; MBL-negative *Enterobacterales*, *n* = 1. Percentages not given when < 10 isolates. MIC_90_ and MIC range data for tigecycline are calculated for all *Enterobacterales* collected

Of the *Enterobacterales*, 1.3% (21/1639) were DTR and 25.0% (410/1639) were MDR. Among MDR isolates, susceptibility rates were highest to ceftazidime-avibactam (96.8%), colistin (94.4%) and tigecycline (100%, *E. coli* and *C. koseri* only), and among DTR isolates, ≥ 57.1% were susceptible to amikacin, ceftazidime-avibactam and colistin (Table 3). Few isolates were susceptible to ceftazidime alone (MDR, 1.2%; DTR, 0.0%).

Of the 30/1639 isolates that were MEM-R, 66.7% were susceptible to amikacin, 70% to ceftazidime-avibactam and 93.3% to colistin; however, only 3.3% were susceptible to ceftazidime alone. Of the 20 MEM-R, MBL-negative isolates, 95.0% were susceptible to ceftazidime-avibactam, 90.0% were susceptible to colistin and only one isolate was susceptible to ceftazidime alone. Ten MEM-R isolates were MBL-positive, of which 9 were amikacin-susceptible and all 10 were colistin-susceptible (data not shown). Among the 26/30 carbapenemase-positive isolates, 65.4% were susceptible to amikacin, 69.2% to ceftazidime-avibactam and 92.3% to colistin.

## Discussion

Susceptibility among *P. aeruginosa* was highest to amikacin, ceftazidime-avibactam and colistin and among the *Enterobacterales*, to ceftazidime-avibactam, colistin and tigecycline (*E. coli* and *C. koseri* only), followed by meropenem and amikacin. Similar results have been reported for isolates collected in 2012–2015 across Europe [10, 11], although for colistin and tigecycline, susceptibility rates among *Enterobacterales* were lower than in our study [11]. This is likely due to inclusion of a broader range of species of *Enterobacterales* by Kazmierczak et al. [11]. Similar ATLAS data were also reported for Central Europe/Israel (2014–2018) [12], indicating that susceptibility rates to ceftazidime-avibactam, colistin and amikacin remain stable in the region. As previously reported [10, 11], susceptibility rates to ceftazidime alone were low compared with ceftazidime and avibactam combined, particularly among resistant subsets.

Among *P. aeruginosa* and *Enterobacterales* 5.7% and 1.3% were DTR, respectively. DTR is a valuable category, comprising isolates that are not susceptible to first-line, high-efficacy, low-toxicity agents [13]. The majority of DTR isolates in our study were susceptible to colistin (*P. aeruginosa*, 100%; *Enterobacterales*, 95.2%) and most DTR *Enterobacterales* were susceptible to ceftazidime-avibactam (76.2%); however, the rate was reduced against DTR *P. aeruginosa* (37.5% susceptible). Amikacin susceptibility rates against DTR isolates were 62.5% (*P. aeruginosa*) and 57.1% (*Enterobacterales*).

Most (82.4%) MEM-R *P. aeruginosa* were MBL-negative and, as with the other subsets in this analysis, their susceptibility was highest to ceftazidime-avibactam, amikacin and colistin. The susceptibility breakpoint for ceftazidime alone only applies at increased exposure, and susceptibility was low compared with ceftazidime-avibactam (29.1% vs. 78.6%), demonstrating the value of combining avibactam with ceftazidime. The other MEM-R isolates (17.6%) were MBL-positive, against which only colistin was active. A lower rate of *Enterobacterales* than *P. aeruginosa* were meropenem-resistant (1.8% vs. 17.8%), similar to the rates reported by Kristóf et al. [12]. Two thirds of MEM-R *Enterobacterales* were MBL-negative and, as reported previously [3], most were susceptible to ceftazidime-avibactam and colistin. As with *P. aeruginosa*, few *Enterobacterales* isolates were susceptible to ceftazidime alone, in line with previous reports [3], again demonstrating the value of the combination.

Overall, 55 (7.8%) *P. aeruginosa* isolates were resistant to ceftazidime-avibactam, similar to that reported for European isolates collected in 2012–2015 [10]. Of these, 23/55 were identified as carbapenemase producers (22 MBL-positive [7 IMP, 15 VIM] and 1 carbapenemase-positive [GES] but MBL-negative). No other GES-positive isolates were identified and for the remaining 32 isolates, no carbapenemase or MBL genes were detected. In contrast, 13 (0.8%) *Enterobacterales* isolates were identified as resistant to ceftazidime-avibactam and 12/13 isolates were MBL-positive (4 VIM, 8 NDM-1; *Citrobacter freundii* [*n* = 1], *Enterobacter cloacae* [*n* = 8] and *K. pneumoniae* [*n* = 3]). For the remaining isolate (*E. coli*), no carbapenemase or MBL genes were detected. Ceftazidime-avibactam is known to be inactive against MBL-producing isolates [3].

There are limitations to this analysis; the study collected a predetermined number of isolates from each centre and so cannot be considered epidemiological. With only 1 year of data, some isolate numbers are low, particularly in the resistance subsets, meaning that some of the data should be treated with some caution.

In conclusion, rates of susceptibility to ceftazidime-avibactam were high among isolates of *P. aeruginosa* and *Enterobacterales* collected from Croatia, Czech Republic, Hungary, Poland, Latvia and Lithuania in 2019 and were similar to activity reported in previous years for isolates collected in Europe. Amikacin and colistin also continue to be active against these Gram-negative isolates, as does tigecycline against isolates of *E. coli* and *C. koseri*. Meropenem susceptibility rates were high among *Enterobacterales* isolates but reduced against *P. aeruginosa*. Ceftazidime-avibactam continues to be a good choice for the treatment of MDR Gram-negative infections, it has a safety profile consistent with that previously observed for ceftazidime alone [14–17] and does not require therapeutic drug monitoring.

## Data Availability

The datasets generated and/or analysed during the current study are available from the corresponding author on reasonable request. Data from the global ATLAS study can be accessed at https://atlas-surveillance.com.
